# A Potential Concept In The Management of Tumors With Modulation of Prostaglandin, Nitric Oxide and Antioxidants

**DOI:** 10.1100/tsw.2007.89

**Published:** 2007-03-30

**Authors:** Noori S. Al-Waili

**Affiliations:** Al-Waili ‘s Foundation for Science and Trading, New York City, NY, USA

**Keywords:** prostaglandins, nitric oxide, cancer, NSAIDs, anemia, immunity, inflammation

## Abstract

Prostaglandins (PGs), nitric oxide (NO), free radicals and chronic inflammation play a major role in tumorogenesis. We have found *in vivo* that PGs suppress antibody production; reduce serum iron, and modulate bone marrow function. Tumors are associated with immunosuppression and anemia. We have hypothesized that the over-production of PGs is responsible for immunosuppression and anemia in conditions associated with increased production of PG such as tumor, and that PG inhibitors might help reversing immunosuppression and anemia, and play a role in eradication and prevention of tumors. This is supported by reports that demonstrate the immunosuppressive effects of PGs in tumors. PG inhibitors have also been shown to be crucial in the prevention of tumors such as esophageal and colon cancers. Others and we have found that high NO production was encountered in patients with cancer while antioxidants are decreased. Evidence supports the efficacy of PG inhibition in malignancies, and the concept of PG inhibition, NO modulation, anti-oxidants, immunotherapy with antibody or immune cells, and anti-inflammatory agents when used in the prevention and management of malignancies are discussed.

## INTRODUCTION

*In-vitro* studies have shown that the addition of malignant cells synthesizing large amounts of PGs to spleen cells stimulated with sheep red blood cell (RBC) caused a marked suppression of antibody production in the sheep RBC. The introduction of a PG inhibitor prevented this suppression[1,2]. In 1977, it was proposed that the soluble mediator responsible for such immunosuppression might be PGs. We studied the effects of PGA1 on antibody production against thymus-dependent antigen (Ag) (Sheep RBC) and thymus-independent Ag (*E. coli*) in rabbits. The results had shown that PGA1 causes a marked suppression of antibody production during primary and secondary immune response[3,4]. It was hypothesized that PG’s might be responsible for immunosuppression encountered in conditions associated with increased PG production (Figure 1). We also studied effects of PGs on hematological and biochemical parameters under various stresses to further understand how the release of large amounts of

PG is involved in the pathogenesis and alteration of these parameters in malignancies. The effect of PG's on serum protein, albumin and globulin during antigenic stimulation was studied[5]. PGA1 causes a significant reduction in serum albumin and changes in serum total protein. It was postulated that the low serum albumin associated with various chronic or acute illnesses might be related to the high PG synthesis associated with these conditions, and inhibition of PGs might be useful in treatment and prevention of malignancies[5,6].

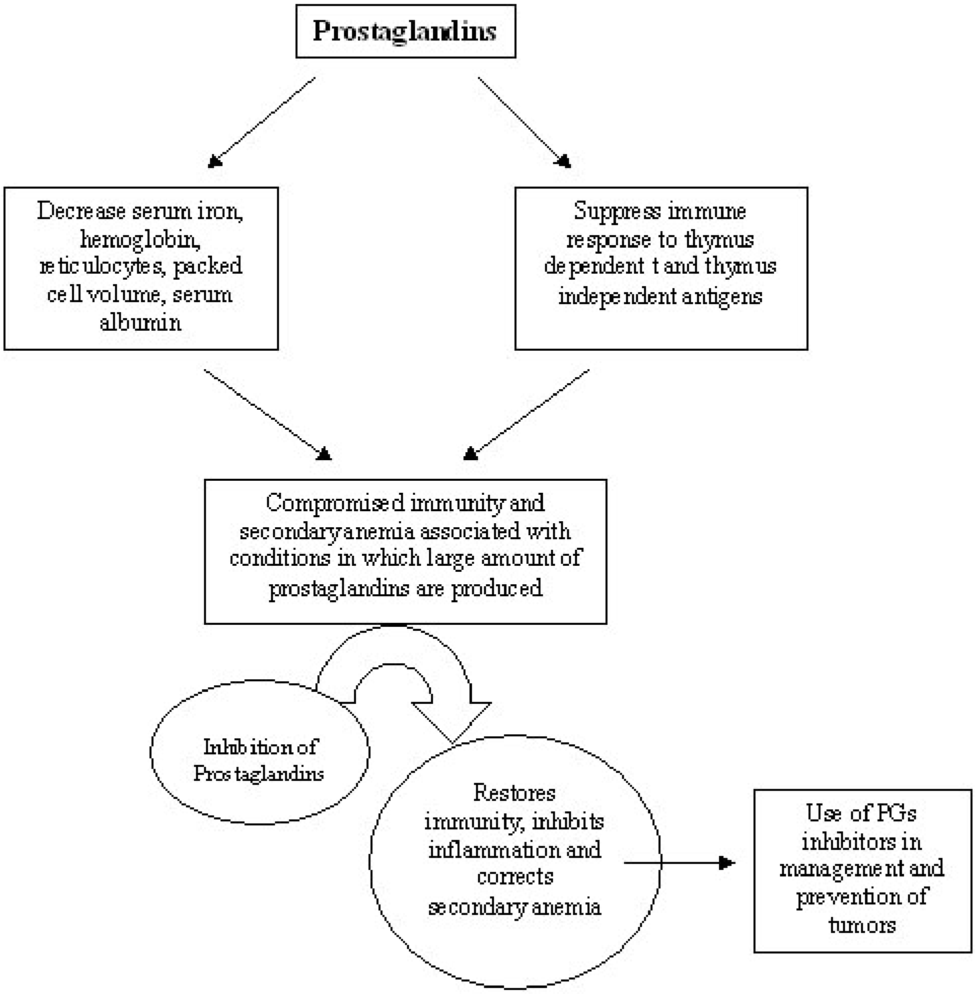

Figure 1: Proposed concept for etiology of immunosuppression and secondary anemia and their possible correction with use of prostaglandin inhibitors

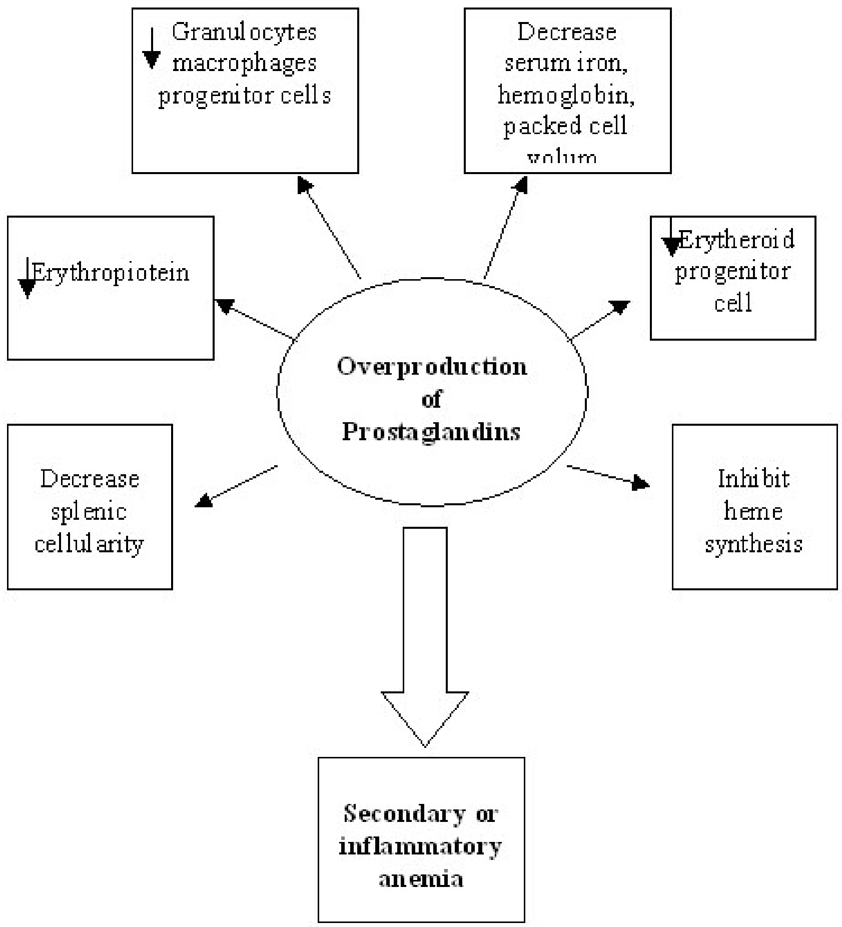

Figure 2- The concept for secondary anemia caused by overproduction of prostaglandins

The effects of PG's on hemopoiesis and hematological indices have been studied during acute and chronic blood loss[7,8,9]. PGE2 caused significant lowering of serum iron and a greater reduction in hemoglobin, RBC and packed cell volume, and decreased reticulocyte counts after acute or chronic blood loss. PGE2 decreased leukoid series after acute blood loss[8]. Following chronic blood loss, PGE2 caused a marked depression of promelocyte, myelocyte, metamyelocyte, segmented cells and total myeloid in bone marrow in comparison to normal and chronically bled animals[9]. It was postulated that PG's might be involved in pathogenesis of secondary anemia, and that PG inhibition might be of value in the management of anemia resulting from malignancies (Figure 2)[10].

The effect of PGF2 alpha on blood sugar was studied; PGF2 alpha causes significant lowering of fasting blood sugar in normal rabbits (11). PGE2 ameliorates hyperglycemia that followed bleeding and ether anesthesia[12]. Similar results were obtained with use of PGE2[13]. PGE2 causes significant suppression of hyperglycemia and elevation in alkaline phosphates following bleeding; and inorganic phosphorous was mildly lowered by PGE2[14]. Therefore, it was postulated that changes in calcium, inorganic phosphorous or blood sugar might be related to increased PG by cancer cells. Clinically, we showed the beneficial effects of PG inhibitor, indomethacin, in the management of chorionic carcinoma and basal cell carcinoma[10,15].

PGs have considerable effects on immune response. In late 1970's and early 80's, *in-vitro* studies demonstrated the immunosuppressive effect of PGs[16,17,18,19]. PGE inhibits human T-cell proliferation and lymphokine production, and suppresses IL-2 production[20,21]. PGE2 exerts selective regulatory effects on human B-cell responses[22]. PG's stimulate suppressor cells to release suppressor lymphokines[23]. PG's inhibit the murine natural killer-cell activity[24]. PGE2 has many immunoregulatory functions including the inhibition of Ia expression, IL1 and TNF production by Mphils and Ag induced T-cell proliferation, the promotion of suppressor T-cell differentiation, and immunoglobulin secreting cells[25]. PGE2 reduces expression of type 1 immune responses such as a contact hypersensitivity reaction[26]. PGE2 has significant immunosuppressive effects on cell-mediated immunity[27]. PGE1 and E2 have immunosuppressive effects on T-cell and Mphil functions. PGE2 enhances the cellular spread of herpes virus probably by suppressing natural killer-cell activity[28].

## Prostaglandins, immunity and Cancer

Evidence from clinical and preclinical studies indicates that PGs participates in carcinogenesis, inflammation, immune response suppression, apoptosis inhibition, angiogenesis, and tumor cell invasion and metastasis[29]. Studies demonstrate defective Ag presentation, T-cell defects, and premature thymic atrophy in cancer patients and tumor-bearing animals (30). Recent review showed that tumor cells inhibit the physiological function of immune cells, and that NSAIDs restore the immune function[31].

Reports showed that PG's are produced in excess from malignancies and are implicated in the growth and spread of tumors[32,33]**.** The addition of MC16 tumor cells to cultures of cells inhibits immune response in sheep RBC; PG synthetase inhibitors block this inhibition[34]. Findings indicated that probably all tumor cells possess immunosuppressive factor(s), which may account for their apparent lack of immunogenicity and the failure of proper immune responses in the tumor-bearing hosts[35]. PG's can modulate a variety of immunological responses and thereby play an important role in host antitumor immunity[36]. Elevated tumor COX-2 and PGE2 levels have been implicated in angiogenesis, tumor invasion, resistance to apoptosis, and suppression of antitumor immunity[37]. Studies suggest that COX-2 assists in determining and defining the metastatic signaling pathways that promote the breast cancer progression to metastasis[38].

PGE2 can act as a feedback inhibitor for cellular immune processes[39]. COX-2 expression is associated with development of aggressive breast cancer[40]. PGE receptors EP1 are associated with breast cancer development, and selective PGE receptor $EP_1$ antagonists may possess chemo-preventive effects through the induction of apoptosis[41]. The mechanisms of carcinogenesis related to COX and PG include oncogene synthesis and expression, upregulation of COX, accelerated cell replication, failed apoptosis, viral activation, and disruption of signaling pathways, autoimmunity, immunosuppression, angiogenesis and metastasis[42]. Recent review shows that studies indicate that COX-2 produced in breast cancer cells may be vital to the development of osteolytic bone metastases in patients with breast cancer, and that COX-2 inhibitors may be useful in halting this process[43]. Other review discussed impact of overexpression of COX-2 in tumors leading to an increase in PG levels, which affect angiogenesis, inhibition of apoptosis, stimulation of cell growth as well as the invasiveness and metastatic potential of malignant cells[44].

## Prostaglandin inhibitors in cancer and Immunity

It was found that indomethacin blocks IFN-gamma-induced suppression in tumor bearing host Mphils -T cell cultures, blocks the immunosuppression in renal cell carcinoma-bearing mice, and restores normal lymphokine-activated killer cell activity for patients with malignant mesothelioma[45,46,47]**.** Indomethacin increases in Th/Ts cell ratio and an improvement in delayed type hypersensitivity response[48]. Indomethacin can increase tumor cell killing by the reversal of the suppression for many immune functions by PGE2[49]. PG inhibitors enhance mitogen responses both before and after breast cancer radiation therapy[50]. Data showed that indomethacin might be of use in immunotherapy of bladder cancer[51]. Piroxicam, nonselective PG inhibitor and widely used for treatment of inflammatory arthritis, stimulates cellular immune function[52].

Indomethacin stimulates immune response o sheep RBC, as well as on the formation and functional activity of the antigen-induced (by the tolerogenous dose of RBC) T-suppressors[53]. Indomethacin enhances the secondary but not the primary humoral immune response[54]. Aspirin and indomethacin have proven immunomodulatory and anti-angiogenic[55]. Indomethacin treatment could partially restore depressed host defense parameters after cardiopulmonary bypass[56].

Epidemiological evidence has shown a decreased incidence of lung cancer in patients who use NSAIDs[57]. NSAIDs inhibit activity of phosphodiesterases and cyclic GMP-AMP protein kinases, which may be central to cancer initiation and promotion. Flurbiprofen enhances tumor response to radiotherapy and chemotherapy, and prolong survival time after excision of primary tumor[58]. COX-2 inhibitors, Rofecoxib and celecoxib, and nonselective NSAIDs protect against the development of colorectal neoplasia and esophageal cancer[59,60]. Sulindac, PG synthesis inhibitor, causes dramatic regression of colonic adenomas in patients with Familial Polyposis[61]. Further, sulindac and its metabolites induce apoptosis in colonic adenomas *in vivo*[62]. Chronic use of aspirin and NSAIDs might be associated with a reduced risk of gastrointestinal cancers[63,64]. Aspirin and COX-2 inhibition is effective for prevention and treatment of experimental breast cancers[65,66]. NO- NSAIDs showed a more efficient anti-tumor-cell effect and chemoprevention effect than standard NSAIDs[67,68]. Recent review supports that NO-NSADs hold the promise of being safe and effective chemopreventive agents against colon cancer[69]. The recent reports of cardiovascular adverse events in patients treated with selective COX-2 inhibitors has been reviewed and outline for using them with minimized side effects are discussed[70].

## Prostaglandin and Prostaglandin Inhibition in Anemia

PGE inhibits the proliferation of normal colony-forming cells[71]. Both COX isoforms are present and active during human erythropoiesis, and COX-derived prostanoids may play a role in erythroid maturation[72]. PG's mediate the suppression of early and late erythroid progenitor cells in patients with chronic renal failure[73]. PGF2 alpha inhibits extrarenal erythropoietin production[74]. Intravenous injection of native PGE2, or 16,16 dimethyl-PGE2 suppressed absolute number of detectable granulocyte-Miph progenitor cells per femur or spleen[75,76]. Myeloid suppressor cells from tumor bearing mice produce suppressive levels of PGE[77]. Co-culture of Ehrlich ascites carcinoma and Sarcoma (S-180) cells with normal mouse bone marrow cells profoundly suppresses formation of MSC colonies[78].

Inhibition of PG with indomethacin increases granulocyte-Mphil progenitors and splenic erythropoiesis[79]. Indomethacin facilitates hematopoietic recovery following 5-FU administration, and enhances the rate of extrarenal erythropoietin production by reducing the PGF2 alpha titer in the liver. Indomethacin dminishes the myelopoietic suppressive effects associated with IL-1 administration and optimizes its myelopoietic stimulatory capacity[80]. PG inhibitors may have a role in combination with hematopoietic growth factors in accelerating hematopoietic recovery following cytoreductive chemotherapy[81].

## Nitric Oxide, Antioxidants and Cancer

NO plays a central role in the physiology and pathology of diverse tissues including the immune system. Like PG and other biological substance, the levels of NO must be carefully regulated to maintain homeostasis. Appropriate levels of NO derived from iNOS help building effective defense against pathogens. In addition, overproduction of NO has been associated with the pathogenesis of many disorders, including atherosclerosis, inflammatory diseases, neurodegenerative diseases, and cancer. Therefore, depending upon the levels of NO generated, NO could be harmful or beneficial. Despite the apparently conflicting observations, it is evident that NO is involved in cancer pathogenesis.

It was found that NO synthesis represents a significant Miph antitumor mechanism[82]. Very low doses of an NO mimetic can produce significant reduction in this resistance phenotype and, therefore, may have a potential therapeutic role. However, it was found that plasma as well as erythrocyte malondialdehyde and plasma NO level was higher in cervical cancer as compared to healthy controls, while antioxidant enzymes activities were decreased[83]. I have found that NO end product increased markedly in patients with breast cancer, and this elevation was correlated with stage of the disease and whether the patients treated or not[84]. Further, antioxidants are decreased in patients with cancer. Leukocyte nitrate and nitrite level was increased and superoxide dismutase activity was decreased in patients with gastric cancer[85]. Lipid peroxidation products, including lipid hydroperoxide and malondialdehyde, NO products, were significantly elevated with a concomitant depletion of antioxidants in head and neck squamous cell carcinoma[86].

Cells are endowed with an antioxidative defense system, which when compromise will lead to pathogenesis of disease including cancer[87]. Therefore, the imbalance between free radicals and antioxidants is the key. Studies suggest that there is a link between the initial involvement of oxidative stress and subsequent induction of the COX mediated inflammatory process. Free radicals are generated in the course of PG synthesis and inhibitors of PG synthesis are free radical scavengers. COX inhibitors decrease superoxide anion production[88]. PGH synthase-dependent co-oxidation of chemicals can result in the intracellular formation of free radical metabolites[89]. Therefore, PG and free radicals potentiate each other, and they involve in malignancies.

Antioxidants neutralize free radicals as the natural by-product of normal cell processes. The oxidative damage can affect the DNA and cell membranes, making the cells more vulnerable to cancer. In both laboratory and animal studies, antioxidants have been shown to protect against this damage.

## Cancer and Inflammation

Oxidative stress and inflammation contribute to carcinogenesis. Angiogenesis is demand for tumor growth and dissemination; inflammation can promote tumor angiogenesis. Studied showed that PGs produced by COX-2 promote tumor development by stimulating cell proliferation and angiogenesis and by suppressing programmed cell death and immune defense[90]. Recent review showed that population-based studies examining individuals with chronic inflammation revealed that suppressed cellular immunity, in combination with enhanced humoral immunity and humoral immunity-associated cytokines, suppress anti-tumor immune responses while simultaneously enhancing angiogenesis and presumably overall cancer risk in afflicted tissue[91]. Accumulation of free radicals leads to inflammation[92]. Chronic inflammation may cause cancers of different organs including stomach, colon, breast, skin, prostate, and pancreas[93,94,95]. A proinflammatory mediators, such as cytokines, chemokines, PGs, NO, and leukotrienes, promote neoplastic transformation of cells by altering normal cellular signaling cascades[96]. Laboratory and epidemiological studies suggest that prolonged use of NSAIDs reduces the risk of malignancies that frequently occur in persistently inflamed tissues[97,98].

## POTENTIAL APPROACH FOR CANCER ETIOLOGY AND THERAPY

Every substance works in coordination with others. Therefore, every biological substance must be within limit to allow it to work with other substances as one unit (Surely we have created everything according to a measure, *Holy Quran*, *Surat Moon*, *version 49*). Lord created every thing in a measure and in purpose. Any sustained changes out of biological balance will cause disturbance and disease. PG and NO are very active substances with wide variety of biological influences, and their disturbed measure could affect tremendously the biological systems.

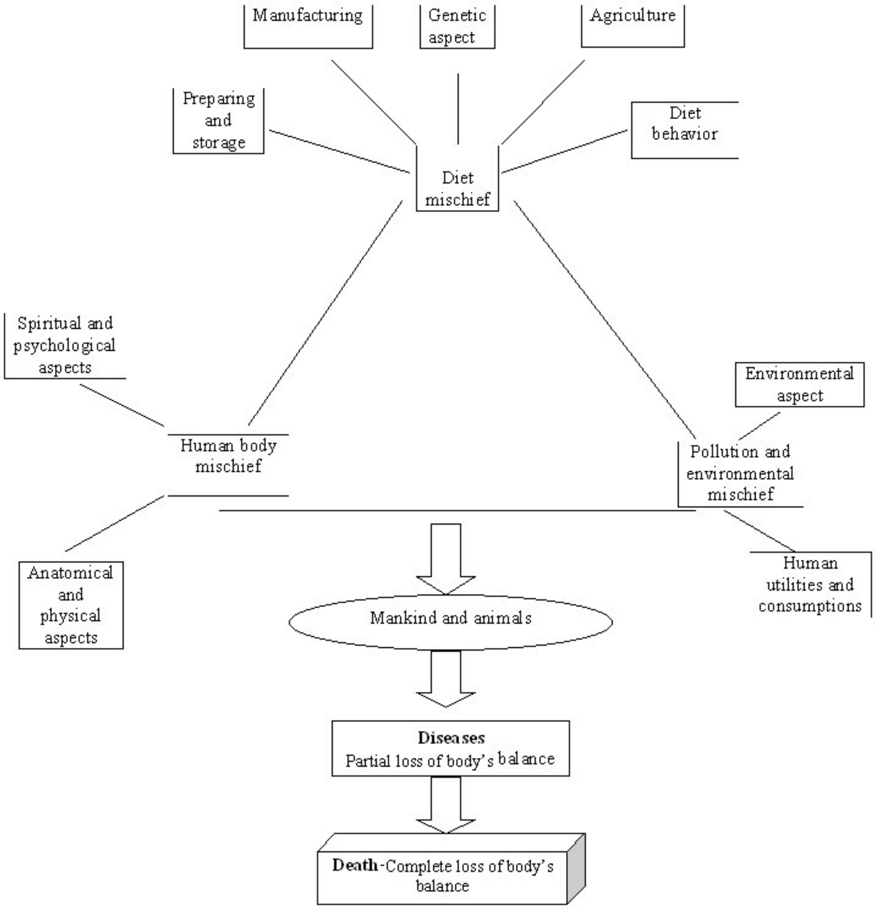

Figure 3: Al-Waili' Disease's Triangle (Basic etiology of illnesses as malignancies)

When immunity, such as humoral immunity, cellular immunity, hormonal balance, enzymatic activity, spiritual attitude, and antioxidants, is disturbed, dividing cells become out of the biological balance and begin to divide and develop into more and more cells, and will cluster into tumors. Defects in the balanced biological functions, caused by the aspects mentioned in Disease's Triangle (Figure 3), should take place before the development of cancer[99,100]. Cancer cells will not grow and spread in healthy body with a balanced biological function. Many mechanisms are proposed for initiation and growing of cancer, but I think that all proposed mechanisms rose as a result of impaired body immunity. Immunity is not humoral or cellular, innate or acquired tools against biological invaders, but it means the power of body that designed to repair imbalance and impairment[12]. Understanding the mechanisms and discovering the mediators that enable these cells to thrive is crucial in learning how to deal with them by activating the immune system.

We found that PGs suppress the lymphoid system, and reduce blood indices, serum iron and serum albumin, and modulate blood glucose, and calcium and phosphorus blood level. Therefore, it was hypothesized that PG's are involved in the immunosuppression and secondary anemia encountered in conditions in which large quantities of PG's are released such as tumors, and PG inhibitors may reverse the immunosuppression and enhance the immune response to tumor growth. This concept allowed using PGs inhibitors for treatment and prevention of cancer. This has been supported by many studies showing that PG's are potent immunosuppressive and administration of PG inhibitors could improve immune function. Long-term use of PG inhibitors decreases the incidence of certain malignancies, including colorectal, esophageal, breast, lung, and bladder cancers. Piroxicam, indomethacin, aspirin and ibuprofen could stimulate immune response or restore immuno-deficiency in certain pathological conditions[27,49,53,54,55,56].

Others and we have found that NO is increased markedly in malignancies and could prompt cancer development. NO-NSAIDs, has have been developed to reduce side effects of NSAIDS, cause cell cycle perturbations and induce apoptosis in cell lines from different tumors[101,102]. Many natural products, such as honey, could lower PGs and increase moderately NO, and therefore they may represent alternative and safer interventions[103,104,105,106]. Chronic inflammation has been associated with increased risk of human cancer[107]. Inflammation facilitates the initiation of normal cells and their growth and progression to malignancy[108]. Free radicals are involved in the pathogenesis of malignancies too.

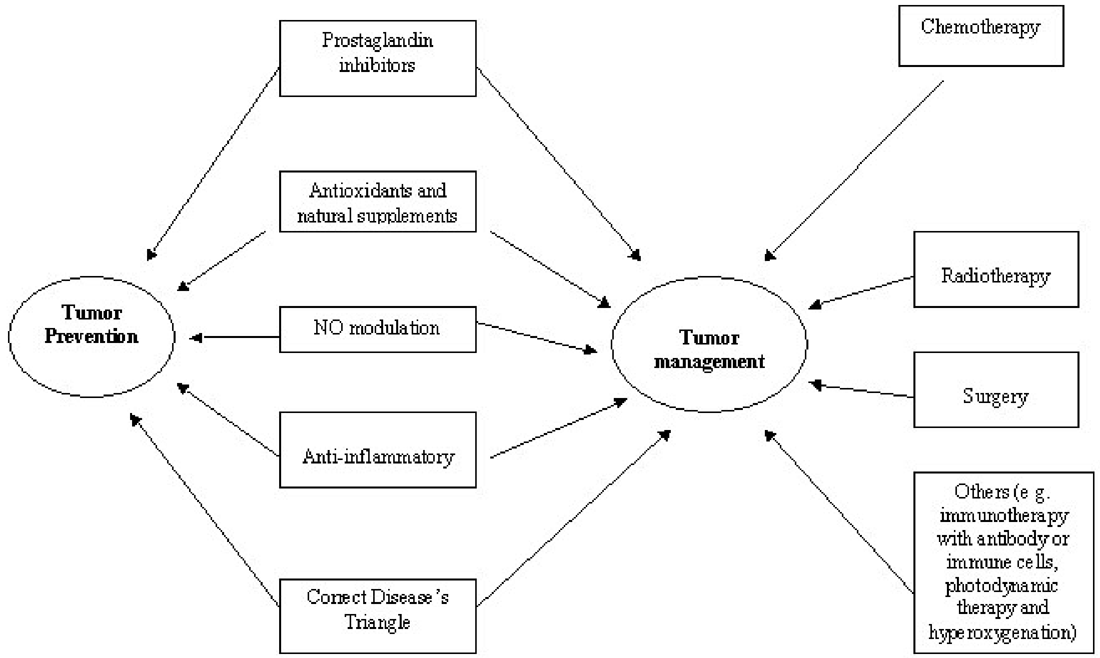

Figure 4: Proposed concept for management of tumors

A recent review discussed the potential use of immunostimulatory monoclonal antibodies directed to immune-receptor molecules in cancer therapy[109]. Gene therapy of targeting specific genetic defects found in certain tumors need extensive work before reaching final conclusion. Using immune cells, such as Miphs, to combat tumor cells and their dissemination warrants further studies and consideration. In this regard we have found that peritoneal cells including Miphs could be transferred intravenously and can combat bacterial infections[110,111,112,113]; these finding pave the way to examining the potential therapeutic effect of peritoneal cells and Miphs for treatment of tumors as well as inflammatory conditions. I believe that the main strategy to prevent and manage malignancies should include inhibition of PG over-production, modulating NO production, use of antioxidant and anti-inflammatory agents, immunotherapy, and improvement of life styles according to the Disease Triangle. These issues could be used alone to help prevent malignancies or, in combination with chemotherapy, radiotherapy and surgical intervention, to manage diagnosed cases of tumors (Figure 4).

## ACKNOWLEDGEMENT

This study and all Al-Waili's studies on PG and PG inhibitors are not supported by grant or financial assistant from any drug company or academic or professional establishment. There is no interest of conflict upon publication of this article.

## RE.FERENCES

1. Otto J. (1975) Subversion of immune response by tumor cells and role of prostaglandin. *Pro Nat Acad Sci* USA **72**, 1848-1852.
2. Zemecki, M. and Webb, D. (1996) Regualtion of immune response to T-independent antigen by prostaglandins and B cells. J Immunol. **117**, 2158-2164.
3. Al-Waili, N., Thewani, A., and Al-Azzawi, H. The effects of PGA1 on antibody production. The World Conference on Clinical Pharmacology and Therapeutics. 1980, London, pp 0246.
4. Al-Waili, N., Thewani, A., and Al-Azzawi, H. (1981) Prostaglandin A1 and antibody production. *J F Med Baghdad*, **23**, 32-35.
5. Al-Azzawi H, Al-Waili N, Thewani A. (1981) The effects of PGAl on serum protein components during primary and secondary Immune responses. *J F Med Baghdad*, **23**, 54-60.
6. Al -Waili N, Three cases of nephrotic syndrome treated by indomathacin, J Pak Med Ass, 1988, 28, 54-56.
7. Al-Waili, N. and Al-Azzawi, H. (1985) The effects of prostaglandin E2 on serum Iron following acute and chronic blood loss. *Clin Exp Pharm Physiol.*, **12**, 443-446.
8. Al-Waili, N., Al-Azzawi, H., and Al-Niami, M., (1983) Bone marrow cellular elements and peripheral blood indices following acute hemorrhage and prostaglandin E2 treatment. *Saudi Med J*, **4**, 236-242.
9. Al-Azzawi, H. and Al-Waili, N., (1984) Haemopoietic changes following chronic blood loss and PGE2 treatment. First Scientific Congress, College of Medicine, University of Baghdad., 109.
10. Al-Waili, N., Al-Azzawi, H., Al-Rawi, Z., (1984) Treatment of advanced chorionic carcinoma by indomethacin and steroids. *Saudi Med J* , **5**, 81-86.
11. Al-Waili, N., Al-Azzawi, H., and Al-Obidi, S. (1981) Hypoglycaemic effect of prostaglandin F2 alpha in normal rabbits. *J F Med Baghdad*,, **25**, 347-249.
12. Al-Waili, N. and Al-Azzawi, H., (1981) The effects of prostaglandin E2 on hyperglycemia following hemorrhage and ether anesthesia. *J F Med Baghdad*, **23**, 405-407.
13. Al-Azzawi, H., Al- dabbagh, A., and Al-Waili, N. (1988) The effects of PGF2 on hyperglycemic response to ether anesthesia and bleeding, The Fourth Scientific Congress, College of Medicine, University of Baghdad, pp.34.
14. Al-Azzawi, H. and Al-Waili, N. (1981) Serum calcium, serum inorganic phosphorus and serum alkaline phosphates during hemorrhage and prostaglandin E2 treatment. *J F Med Baghdad*, **23**, 349-352.
15. Al-Waili, N. (1989) Indomethacin in basal cell carcinoma. *JPMA* **39**, 134-136.
16. Pelus, L. and Straussen, H. (1977) Prostaglandins and the immune response. *Life Sci.* **20,** 903-905.
17. Goodwin, S. and Webb, D. (1980). Regulation of the immune response by prostaglandins. *Clin. Immunol. Immunopathol* **15**, 106-110.
18. Baker, P., Fahey, J., and Munck, A. (1981) Prostaglandin inhibition of T-cell proliferation is mediated at two levels. *Cell Immunol* **61,** 52–61.
19. Kato, K. and Askenase, P. Reconstitution of an inactive antigen-specific T cell suppressor factor by incubation of the factor with prostaglandins. *J Immunol* **133**, 2025–2031.
20. Goodwin, J.S., Messner, R.P. and Peake, G.T. (1978) Prostaglandin suppression of mitogen-stimulated lymphocytes in vitro.J. Clin Invest 1978; 62:753-756.
21. Gordon, D., Bray, M., and Morley, J. (1976) Control of lymphokine secretion by prostaglandins. *Nature* **262**, 400-403.
22. Thompson, P., Jelinek, D., and Lipsky, P. (1984) Regulation of human B cell proliferation by prostaglandin E2. *J Immunol.* **133**, 2446-2453.
23. Webb, D., Wieder, K., Rogers, T., Healy, C., and Nowowiejski-Wieder, I. (1985) Chemical identification of a prostaglandin-induced T suppressor. *Lymphokine Res*.**4**, 139–149.
24. Brunda, M., Herberman, R., and Holden, H. (1980) Inhibition of murine natural killer cell activity by prostaglandins. *J Immunol.* **124**, 2682-2687.
25. Alvarellos, A., Lipsky, P., and Jasin, H. (1988) Prostaglandin $E_2$ modulation of rheumatoid factor synthesis. *Arthritis Rheum* **31**, 1473-1480.
26. Hart, P., Townley, S., Grimbaldeston, M., Khalil, Z., and Finlay-Jones, J. (2002) Mast cells, neuropeptides, histamine, and prostaglandins in UV-induced systemic immunosuppression. *Methods*.**28**, 79-89.
27. Josten, C., Griga, T., and Muhr, G. (1991) Immunostimulation with ibuprofen in chronic osteitis. An experimental study. *Unfallchirurg* **94**, 191-193.
28. Milch, P., Salvatore, W., Luft, B., and Baker, D. (1988) Suppression of newborn natural killer cell activity by prostaglandin E2. *Am J Obstet Gynecol* , **159**, 47-51.
29. Choy, H. and Milas, L. (2003) Enhancing radiotherapy with cyclooxygenase-2 enzyme inhibitors: a rational advance? *J Natl Cancer Inst.* **95**, 1440-1452.
30. Ohm, J. and Carbone, D. (2002) Immune dysfunction in cancer patients. *Oncology (Huntingt)* **16(1 Suppl 1)**, 11-18.
31. Lang, S., Picu, A., Hofmann, T., Andratschke, M., Mack, B., Moosmann, A., Gires, O., and Tiwari, S. (2006) COX-inhibitors relieve the immunosuppressive effect of tumor cells and improve functions of immune effectors. *Int J Immunopathol Pharmacol.* **19**, 409-419.
32. Karim, S. and Raq, B. (1976) Prostaglandins and tumors. In: Karim SMM,ed. Prostaglandins: physiolocal, pharmacological and pathological aspects. Lancaster: MTP Press, 1 pp. 145-178.
33. Bennett, A., Charlier, E., and MacDonald, A. (1979) Bone destruction by breast cancer. *Prostaglandins* **11**, 461-469.
34. Grinwich, K. and Plescia, O. (1977) Tumor-mediated immunosuppression: prevention by inhibitors of prostaglandin synthesis. *Prostaglandins.* **14**, 1175-1178.
35. Stichtenoth, D., Gutzki, F., Tsikas, D., Selve, N., Bode-Boger, S., Boger, R., and Frolich, J. (1994) Increased urinary nitrate excretion in rats with adjuvant arthritis. *Ann Rheum Dis.* **53**, 547-549.
36. Eddleston, M., Karalliedde, L., Buckley, N., Fernando, R., Hutchinson, G., Isbister, G., Konradsen, F., Murray, D., Piola, J., Senanayake, N., Sheriff, R., Singh, S., Siwach, S., and Smit, L. (2002) Pesticide poisoning in the developing world--a minimum pesticides list. *Lancet* **360,** 1163-1167.
37. Sandler, A., Dubinett, S. (2004) COX-2 inhibition and lung cancer *Semin Oncol.* **31(2 Suppl 7)**, 45-52.
38. Larkins, T.L., Nowell, M., Singh, S., (2006) Sanford, G.L. Inhibition of cyclooxygenase-2 decreases breast cancer cell motility, invasion and matrix metalloproteinase expression. *BMC Cancer.* **6**, 181-185.
39. Eddleston M, Karalliedde L, Buckley N, Fernando R, Hutchinson G, Isbister G, Konradsen F, Murray D. Pesticide poisoning in the developing world--a minimum pesticides list. *Lancet.* **360**, 1163-1167.
40. Arun, B. and Goss, P. (2004) The role of COX-2 inhibition in breast cancer treatment and prevention. *Semin Oncol.*, **31(2 Suppl 7)**, 22-29.
41. Ishihara, S., Rumi, A., Okuyama, T., and Kinoshita, Y. Effect of prostaglandins on the regulation of tumor growth. *Curr Med Chem Anti-Canc Agents.* **4**, 379-387.
42. Lieb, J. (2001) Antidepressants, eicosanoids and the prevention and treatment of cancer. A review. *Prostaglandins Leukot Essent Fatty Acids.* **65**, 233-239.
43. Singh, B., Berry, J.A., Shoher, A., Ayers, G.D., Wei, C., and Lucci, A. (2007) COX-2 involvement in breast cancer metastasis to bone. *Oncogene.* Jan 8
44. Cervello, M. and Montalto, G. Cyclooxygenases in hepatocellular carcinoma. *World J Gastroenterol.* **12**, 5113-5121.
45. Alleva, D., Burger, C., and Elgert, K. Tumor growth causes suppression of autoreactive T-cell proliferation by disrupting macrophage responsiveness to interferon-gamma. *Scand J Immunol.* **39**, 31-38.
46. Gregorian, S. and Battisto, J. (1990) Immunosuppression in murine renal cell carcinoma. II. Identification of responsible lymphoid cell phenotypes and examination of elimination of suppression. *Cancer Immunol Immunother* **31**, 335-341
47. Manning, L.S., Bowman, R.V., Davis, M.R., Musk, A.W., and Robinson, B.W. (1989) Indomethacin augments lymphokine-activated killer cell generation by patients with malignant mesothelioma. *Clin Immunol Immunopathol* **53**, 68-77.

48. Gogos, C., Maroulis, J., Zoumbos, N., Salsa, B., and Kalfarentzos, F. (1995) The effect of parenteral indomethacin on T-lymphocyte subpopulations and cytokine production in patients under major surgical operations. *Res Exp Med* **195**, 85-92.
49. Kim, C.D., Sung, M.W., Lee, S.J., Heo, D.S., Yoon, S.J., and Kim, K.H. (1999) The effect of prostaglandin and its inhibitor on antibody-dependent cellular cytotoxicity against human squamous cell carcinoma of head and neck. *Anticancer Res.* **19(1A)**, 455-459.
50. Wasserman, J., Blomgren, H., Rotstein, S., Petrini, B., and Hammarstrom, S. (1989) Immunosuppression in irradiated breast cancer patients: in vitro effect of cyclooxygenase inhibitors. *Bull N Y Acad Med* **65,** 36-44.
51. Wang, Z., Chen, Y., Zheng, R., Qin, D., Chen, X., Wang, Y., and Liu, G. In vitro effects of prostaglandin E2 or indomethacin on the proliferation of lymphokine-activated killer cells and their cytotoxicity against bladder tumor cells in patients with bladder cancer. *Prostaglandins* **54**, 769-779.
52. Earnest, D., Hixson, L., and Alberts, D. Piroxicam and other cyclooxygenase inhibitors: potential for cancer chemoprevention. *J Cell Biochem* **Suppl 161**, 156-166.
53. Martynova, T.V., Aleksieieva, I.M., Bryzhina, T.M., Aleksiuk, L.I., and Sukhina, V.S. (2003) Effect of inhibitors of cyclooxygenase and lypoxygenase pathway of metabolism of arachidonic acid on the development and suppression of the immune response in mice. *Fiziol Zh.* **49(1)**, 18-22
54. Goodwin, S., Selinger, D., Messner, R., and Ree, R. (1978) Effect of indomethacin in vivo on humoral and cellular immunity in humans. *Infect Immun.,* **19,** 430–433.
55. O'Byrne, K.J., Dalgleish, AG, Browning, M.J., Steward, W.P., and Harris, A.L. (2000) The relationship between angiogenesis and the immune response in carcinogenesis and the progression of malignant disease. *Eur J Cancer.* **36(2)**, 151-169.
56. Markewitz, A., Faist, E., Lang, S., Endres, S., Fuchs, D., and Reichart, B. (2004) Successful restoration of cell-mediated immune response after cardiopulmonary bypass by immunomodulation. *J Thor Cardiovascular Surg* **105**, 15-24.
57. Brown, J. and DuBois, R. (2005) COX-2: a molecular target for colorectal cancer prevention. *J Clin Oncol* **23**, 2840-2855.
58. Bennett, A., Berstock, D., and Carrol, M. (1981) Enhanced anticancer effects by combining cytotoxic drugs with the prostaglandin synthesis inhibitor flurbiprofen. *Br J Pharmacol* **74**, 208-212P.
59. Rahme, E., Barkun, N., Toubouti, Y., and Bardou, M. The cyclooxygenase-2-selective inhibitors rofecoxib and celecoxib prevent colorectal neoplasia occurrence and recurrence. *Gastroenterology* **125**, 404-412.
60. Bardou, M., Barkun, A., Ghosn, J., Hudson, M., and Rahme, E. (2004) Effect of chronic intake of NSAIDs and cyclooxygenase 2-selective inhibitors on esophageal cancer incidence. *Clin Gastroenterol Hepatol* **2**, 880-884.
61. Morgan, G. and Vainio, H. (1998) Barrett's oesophagus, oesophageal cancer and colon cancer: an explanation of the association and cancer chemopreventive potential of non-steroidal anti-inflammatory drugs. *Eur J Cancer Prev* **7**, 195-199.
62. Ahnen, D. (1998) Colon cancer prevention by NSAIDs: what is the mechanism of action. *Eur J Surg Suppl* **582**, 111-114.
63. Jiang, X. and Wong, B. (2003) Cyclooxygenase-2 inhibition and gastric cancer. *Curr Pharm Des* **9,** 2281-2288.
64. Piazuelo, E., Jimenez, P., and Lanas, A. (2003) COX-2 inhibition in esophagitis, Barrett's esophagus and esophageal cancer. *Curr Pharm Des* **9,** 2267-2280.
65. Terry, M., Gammon, M., Zhang, F., Tawfik, H., Teitelbaum, S., Britton, J.A., Subbaramaiah, K., Dannenberg, A.J., and Neugut, A. (2004) Association of frequency and duration of aspirin use and hormone receptor status with breast cancer risk. *JAMA.* **291**, 2433-2440.
66. Rao, C. and Reddy, B. (2004) NSAIDs and chemoprevention. *Curr Cancer Drug Targets* **4**, 29-42.
67. Rigas, B. and Kashfi, K. (2004) Nitric-oxide-donating NSAIDs as agents for cancer prevention. *Trends Mol Med* **10** 324–330.
68. Ouyang, N. (2006) Nitric oxide-donating aspirin prevents pancreatic cancer in a hamster tumor model. *Cancer Res.* **66** 4503–4511.
69. Rigas, B. (2007) The use of nitric oxide-donating nonsteroidal anti-inflammatory drugs in the chemoprevention of colorectal neoplasia. *Curr Opin Gastroenterol,* **;23(1)**, 55-59.
70. Al-Waili, N., Salom, K., Al-Waili, T., AL-Waili, A., and AL-Waili, H. (2007) Modulation of prostaglandin activity, Part 1: Prostaglandin inhibition in the management of nonrheumatologic diseases: Immunologic and hematologic aspects. *Adv Ther* (in press).
71. Mendelson, J. and Nordberg, J. (1979) Adenylate cyclase in thymus-derived and bone marrow-derived lymphocytes from normal donors and patients with chronic lymphocytic leukemia. *J Clin Invest* **63**, 1124.
72. Dunn, C. (1981) Prostaglandins and erythropoiesis: structure/action relationships and identification of the prostaglandin responsive cells. *Blut* **42**, 307-314.
73. Taniguchi, S., Shibuya, T., Harada, M., Niho, Y. (1989). Prostaglandin-mediated suppression of in vitro growth of erythroid progenitor cells. *Kidney Int* **36**, 712-718
74. Fried, W., Morley, C., Barone-Varelas, J., Bidani, A., and Prancan, A. (1988) Effect of indomethacin and of prostaglandins on extrarenal erythropoietin production in rats. *J Lab Clin Med* **111**, 184-188.
75. Gentile, P., Byer, D., and Pelus, L. (1983) In vivo modulation of murine myelopoiesis following intravenous administration of prostaglandin E2. *Blood* **62**, 1100-1107.
76. Gentile, P. and Pelus, L. (1987) In vivo modulation of myelopoiesis by prostaglandin E2. II. Inhibition of granulocyte-monocyte progenitor cell (CFU-GM) cell-cycle rate. *Exp Hematol* **15**, 119-126
77. DeGowin, R., Gibson, P., and Knapp, A. (1981) Prostaglandin E and the erythropoietic and stromal insufficiency induced by extramedullary tumor. *J Lab Clin Med* **98**, 217-226.
78. DeGowin, R., Gibson, D., Knapp, S., and Wathen, L. (1981) Tumor-induced suppression of marrow stromal colonies. *Exp Hematol.* **9**, 811-819.
79. Boorman, G.A., Luster, M.I., Dean, J.H., and Luebke, R.W. (1982) Effect of indomethacin on the bone marrow and immune system of the mouse. *J Clin Lab Immunol* **7**, 119-126.
80. Pelus, L. (1989) Blockade of prostaglandin biosynthesis in intact mice dramatically augments the expansion of committed myeloid progenitor cells (colony-forming units-granulocyte, macrophage) after acute administration of recombinant human IL-1 alpha. *J Immunol* **143**, 4171-4179.
81. O'Reilly, M. and Gamelli, R.L. (1990) Indomethacin augments granulocyte-macrophage colony-stimulating factor-induced hematopoiesis following 5-FU treatment. *Exp Hematol* **18**, 974-978.
82. Baker, P., Fahey, J., and Munck, A. (1981) Prostaglandin inhibition of T cell proliferation is mediated at two levels. *Cell. Lmmunol* **61**, 52-56.
83. Beevi, S.S., Rasheed, M.H., and Geetha, A. Evidence of oxidative and nitrosative stress in patients with cervical squamous cell carcinoma. *Clin Chim Acta.* **375(1-2)**, 119-123.
84. Al-Waili, N. (2003) Oxidants and antioxidants in breast cancer: Possible tumor marker. FASEB J 2003; 17:861.
85. Dincer, Y., Akcay, T., Tortum, O.B., and Dogusoy, G. (2006) Nitric oxide and antioxidant defense in patients with gastric cancer. *Dig Dis Sci.***;51(8)**, 1367-1370
86. Rasheed MH, Beevi SS, Geetha A. Evidence of oxidative and nitrosative stress in patients with cervical squamous cell carcinoma. *Clin Chim Acta.* **375(1-2)**, 119-123.
87. Al-Waili, N. (2002) Oxidant stress: A common cause for diseases. *ALSaha Wltab*, **188**, 19-25.
88. DeGaetano, C., Cerletti, G., Dejana, E., and Latin, R. (1985) Pharmacology of platelet inhibition in humans: implications of the salicylate-aspirin interaction. *Circulation* **72**, 1185-1193.
89. Schreiber, J., Mason, R., and Eling, T. (1986) Detection of glutathione free radical catalyzed by prostaglandin hydroperoxidase present in intact epidermal cells. *Fed Proc Fed Am Soc Exp Biol.* **45,** 70-75.
90. Marks, F., Furstenberger, G., and Muller-Decker, K. (2007) Tumor promotion as a target of cancer prevention. *Recent Results Cancer Res.* **174**, 37-47
91. Tan, T.T. and Coussens, L.M. (2007) Humoral immunity, inflammation and cancer. *Curr Opin Immunol.* Feb 1
92. Symons, A.M. and King, L.J. (2003) Inflammation reactive oxygen species and cytochrome P450. *Inflammopharmacology*, **11**, 75-86.
93. O'Byrne, J. and Dalgleish, G. (2001) Chronic immune activation and inflammation as the cause of malignancy. *Br J Cancer* **85**, 473-483.
94. Nelson, G., De Marzo, M., DeWeese, T.L., and Isaacs, B. (2004) The role of inflammation in the pathogenesis of prostate cancer. *J Urol* **172**, S6-S11.
95. Whitcomb, C. (2004) Inflammation and Cancer V. Chronic pancreatitis and pancreatic cancer. *Am J Physiol.* **287**, G315-G319.
96. Coussens, L.M. and Werb, Z. (2002) Inflammation and cancer. *Nature.* **420**, 860-867.
97. DuBois, R.N. and Smalley, W.E. (1996) Cyclooxygenase NSAIDs and colorectal cancer. *J Gastroenterol.* **31**, 898-906.
98. Ishikawa, H. (2004) Chemoprevention of carcinogenesis in familial tumors. *Int J Clin Oncol* **9**, 299-303.
99. Mannie, M., Prevost, K., and Marinakis, C. (1995) Prostaglandin E2 promotes the induction of anergy during T helper cell recognition of myelin basic protein. *Cell Immunol.* **160**, 132-138
100. Thompson, P., Jelinek, D., and Lipsky, P. (1984) Regulation of human B cell proliferation by prostaglandin E2. *J Immunol.* **133**, 2446-2453.
101. Spiegel, A., Hundley, T.R., Chen, J., Gao, J., Ouyang, N., Liu, X., Go, M.F., Tsioulias, G.J., Kashfi, K., and Rigas, B. (2005) NO-donating aspirin inhibits both the expression and catalytic activity of inducible nitric oxide synthase in HT-29 human colon cancer cells. *Biochem Pharmacol.* **70**, 993-1000.
102. Fabbri, F., Brigliadori, G., Ulivi, P., Tesei, A., and Vannini, I. (2005) Pro-apoptotic effect of a nitric oxide-donating NSAID, NCX 4040, on bladder carcinoma cells. *Apoptosis.* **10,** 1095-1103.
103. Al-Waili, N. and Boni, N. (2003) Natural honey lowers plasma prostaglandin concentrations in normal individuals. *J Med Food* **6**, 129-133.
104. AL-Waili, N. (2005) Effect of honey on urinary prostaglandin and total nitrite. *Int Nephrol Urol* **37**, 107-111
105. Al-Waili, N. and Boni, N. (2004) Honey increased saliva, plasma, and urine content of total nitrite concentrations in normal individuals. *Med Food* **7**, 377-780.
106. Al-Waili, N. (2003) Identification of nitric oxide metabolites in various honeys and effect of honey on plasma and urinary nitrite/nitrate concentrations. *J Med Food* **6**, 359-364.
107. Garcea, G., Dennison, A.R., Steward, W.P., and Berry, D.P. (2005) Role of inflammation in pancreatic carcinogenesis and the implications for future therapy. *Pancreatology.* **5(6)**, 514-529.
108. Ohshima, H., Tatemichi, M., Sawa, T. (2003) Chemical basis of inflammation-induced carcinogenesis. *Arch*

*Biochem Biophys.* **417**, 3-11

109. Melero, I., Hervas-Stubbs, S., Glennie, M., Pardoll, D.M., and Chen, L. (2007) Immunostimulatory monoclonal antibodies for cancer therapy. *Nat Rev Cancer.* **7**, 95-106
110. AL-Waili, N. (1989) Peritoneal macrophages transfusion in the treatment of chronic postoperative wound infections. *J Pak Med Assoc.* **39**, 310-312.
111. Al-Waili, N. (1986) Allogeneic transfusion of macrophages in acute urinary tract infection. *Clin Exp Pharmacol Physiol.* **13**, 173-176
112. Fakhri, O., Al-Waili, N., Al-Azawi, H., and Makkiya, A. (1984) A note on xenogeneic transfusion of macrophages in experimental septicaemia. *J Appl Bacteriol.* **57**, 531- 533
113. Al-Waili, N. (1988) Xenogeneic transfusion of macrophages in experimental septicemia. *Trans Sci* **10**, 324-326
